# Police–suspect interactions and confession rates are affected by suspects’ alcohol and drug use status in low-stakes crime interrogations

**DOI:** 10.3389/fpsyg.2022.983362

**Published:** 2022-09-15

**Authors:** Angelica V. Hagsand, Hanna Zajac, Lovisa Lidell, Christopher E. Kelly, Nadja Schreiber Compo, Jacqueline R. Evans

**Affiliations:** ^1^Department of Psychology, University of Gothenburg, Gothenburg, Sweden; ^2^Department of Sociology and Criminal Justice, Saint Joseph’s University, Philadelphia, PA, United States; ^3^Department of Psychology, Florida International University, Miami, FL, United States

**Keywords:** police, interrogations, suspects, alcohol and drug-related crimes, taxonomy of interrogation methods framework, Sweden, low-stakes crimes

## Abstract

**Background:**

Low-stakes crimes related to alcohol and/or drugs are common around the world, but research is lacking on police–suspect interactions of such crimes. A large proportion of these suspects are intoxicated during interrogations, and many may have substance use disorder, making them potentially vulnerable to interrogative pressure.

**Methods:**

To address this lack of knowledge, the taxonomy of interrogation methods framework (i.e., 60+ interrogation techniques classified into five domains) and a common classification of question types (appropriate vs. inappropriate) were applied in the coding of written police interrogations. Two archival studies, one pilot (Study 1, *N* = 39) and one main study (Study 2, *N* = 97) analyzed police interrogations with suspects of alcohol- and drug-related crimes in Sweden.

**Results:**

For both Study 1 and 2, suspects showed signs of alcohol and/or drug intoxication, hangover or withdrawal in more than 50% of all interrogations. In Study 2, additional coding indicated that suspects displayed signs of substance use disorder in 57% of the interrogations. The main results from both studies revealed a large number of direct questions asked by the police across all interrogations, and relatively little use of the strategic interrogation techniques from the taxonomy of interrogation methods framework. In fact, when it came to interrogation techniques, law enforcement used more confrontational techniques in their interactions with intoxicated suspects compared to sober suspects. Furthermore, suspects displaying signs of substance use disorder were significantly more cooperative and prone to confess than suspects without indicators of substance use disorder.

**Conclusion:**

As the first novel study on low-stakes crime interrogations related to alcohol and/or drugs, the present study provides useful information about current Swedish interrogation practices and areas for improvement. The study results indicate that suspects displaying signs of intoxication or substance use disorder may be more vulnerable during police interrogations. This may in turn have the potential to inform the development of new interrogation policies. Due to the novelty of this research, more studies are needed, both on a national and international level, to examine interrogations in low-stakes crimes further.

## Introduction

What is known about police interrogation from real-world observational research has overwhelmingly been based on interrogations of suspects of serious crimes such as murder and rape (e.g., Kelly et al., 2019; Izotovas et al., 2021; Leahy-Harland and Bull, 2021). Far less is known, however, about police–suspect interactions when crimes are less serious and the stakes are lower, such as in cases related to alcohol and drug misuse and possession. Crimes related to alcohol or narcotics are common worldwide and, according to police surveys, suspects are often intoxicated both when arrested and during interrogation (Evans et al., 2009; Monds et al., 2021; Hagsand et al., 2022a). Intoxication may also make suspects vulnerable in legal contexts (see Mindthoff et al., 2018, 2020, 2022). Further, in Sweden where the present study was conducted, in 2020 a majority (58%) of inmates suffered from substance use disorder (Swedish Prison and Probation Service, 2021) and similar results have been found in other countries such as the United States (National Center on Addiction and Substance Abuse at Colombia University, 2010). There is also evidence that offender drug use at the time of the crime impacts judges’ sentencing decisions, such that these suspects receive longer sentences (Spohn et al., 2014). In relation to this it is important to note that persons suffering from alcohol- and drug-related issues are often stigmatized in society due to peoples’ stereotypical perceptions (e.g., van Olphen et al., 2009; Lloyd, 2012; Dyregrov and Selseng, 2021), making it even more important to examine how police interacts with this suspect group during interrogations.

In Swedish interrogations regarding serious offenses, there is usually a defense attorney present, and the interrogation is audio/video recorded, assuring a certain level of legal protection for the suspect (Swedish code of juridical procedures, 2017:176, 23 chapter. 21§). In contrast, when it comes to minor offenses such as driving under the influence (i.e., DUI) or drug use, these precautions are rarely taken. The decision to audio/video record interrogations is based on the severity of the crime and the *potential* significance of the suspect’s statement for the criminal investigation (Swedish code of juridical procedures, 2017:176, 23 chapter. 21§). This could potentially result in unethical interrogation methods going unnoticed due to lack of electronic recordings (see Kassin et al., 2014). In cases of drug- and alcohol-related offenses this is even more problematic as some suspects may be under the influence of drugs or alcohol (i.e., experiencing ongoing intoxication) and/or suffer from chronic addiction problems, which might be risk factors for increased suggestibility, as well as the risk of false confessions (Pearse et al., 1998; Gudjonsson et al., 2002, 2004, but see Mindthoff et al., 2021). Although interrogations concerning less serious crimes oftentimes are not audio/video recorded, each interrogator is bound by law (Swedish code of juridical procedures, 2017:176, 23 chapter. 21§) to take notes and read their handwritten or computer-typed interrogation notes out loud so that suspects have the chance to approve or disapprove them (with the possibility to correct the interrogation notes before they are finalized). Despite these safeguards, it is nonetheless important to examine how interrogations with intoxicated and/or substance abusing suspects are being carried out as no previous study has examined this matter.

There is surprisingly little archival research on real police interrogation methods with intoxicated suspects and suspects sufferings from substance use disorders. In Sweden, similar to other countries, it is common for police to encounter intoxicated witnesses, victims, and suspects, but how these interrogations are conducted and whether intoxication is taken into account appears to vary, with no scientific evidence-based guidelines for best practice (Hagsand et al., 2022a,b). Given the high prevalence of intoxicated and drug abusing suspects and the lack of policy guidelines for interrogations, the present study examined how signs of intoxication or substance use disorder may be related to police interrogation behavior and interrogation outcomes. To this end, the present research included two archival studies (one pilot study and one main study) of Swedish police interrogations and focused on suspects of low-stakes interrogations in alcohol- and drug-related crimes (i.e., driving under the influence, drug use and/or possession). The aim was to explore interrogation methods in a convenience sample of low-stakes interrogations, in a previously overlooked and potentially vulnerable suspect group. The present research was, to our knowledge, the first archival study to examine police interrogation methods and police–suspect interactions in alcohol- and drug-related crimes.

To date, most research on the potential effects of alcohol on memory of criminal events has concerned witnesses’ memory, and not that of suspects (but see Mindthoff et al., 2022). Both police officers (e.g., Evans et al., 2009; Hagsand et al., 2022b) and expert witnesses (Kassin et al., 2001) have been found to believe that intoxication reduces witness credibility. Results have suggested that alcohol can affect both the quantity and the quality of witness recall under certain conditions, especially with higher intoxication levels (see Altman et al., 2019; Jores et al., 2019, for a literature review and meta-analysis, respectively). According to several witness memory studies, it is also best to interview intoxicated witnesses immediately, rather than wait for them to sober up (e.g., La Rooy et al., 2013; Hagsand et al., 2017; Hildebrand Karlén et al., 2017; Schreiber Compo et al., 2017). However, these results are not necessarily transferable to suspects since suspects are active participants in crimes rather than passive observers, have different goals and strategies than witnesses, and have an interest in withholding information (Gehl and Plecas, 2017). Among the few alcohol studies on mock suspects, there are some emerging findings that alcohol can affect suspects’ memory and cognition. In an experimental study with mock suspects under the influence of alcohol, intoxication impaired the recall of a mock criminal event, especially for information about people (Read et al., 1992); recall of the mock-suspects’ own actions was also impaired. In a quasi-experimental study in real bars by van Oorsouw et al. (2015), alcohol impaired memory in participants carrying out a mock crime, both during an immediate interrogation and later when participants were sober. Furthermore, an experimental lab study examined alcohol-intoxicated participants’ decision-making skills on whether they would report a transgression carried out by themselves or someone else. Alcohol did actually not affect the risk of disclosing transgressions in this particular study – possibly due to the low-to-moderate doses of alcohol administered (Mindthoff et al., 2019). Other than impacting memory and cognition, there is some emerging evidence that intoxication may also increase suggestibility. Suggestibility in suspects has in some cases been shown to lead to false confessions, and according to a recent review, both alcohol and cannabis may increase suggestibility to false memories (Kloft et al., 2021, but see Flowe and Schreiber Compo, 2021; Mindthoff et al., 2021). Further, in a self-report survey of incarcerated persons in Iceland, many offenders were under the influence both during the offense and the interrogation, and they consistently reported that the intoxication had confused them (Sigurdsson and Gudjonsson, 1994). Aside from evidence that intoxication during an interrogation might affect a suspect’s cognition and memory, chronic substance abuse might affect the interrogation even when the suspect is not intoxicated. In a self-report study with students who had experienced being interrogated, substance abuse was considered an important factor associated with false confessions (Gudjonsson et al., 2007). The authors argued that substance abuse, along with other aversive life events, may decrease the ability to cope with stressful situations, such as an interrogation. One reason for substance dependent suspects to falsely confess is that they might experience drug-withdrawal during incarceration and interrogation. This could contribute to both internalized false confession due to increased suggestibility and confabulations in memory recall, and compliant false confession in the hopes of being released and attaining the substance from which they are withdrawing (Gudjonsson et al., 2007). In a field study, applying the *Gudjonsson Suggestibility Scales* (Gudjonsson, 1984, 1987), patients at a rehabilitation clinic, withdrawing from alcohol, were significantly more suggestible than the two ‘control’ groups (discharge group and AA group) (Gudjonsson et al., 2002). Overall, they were also more cognitively impaired and had higher state and trait anxiety scores. However, in an archival United States study of prosecutor case files with alcohol and/or drug intoxicated suspects of serious crimes (i.e., robbery, assault and rape), suspects who were under the influence were just as likely as sober suspects to admit to the crime (Palmer et al., 2013). However, the majority of intoxicated suspects were arrested on the same day as the crime, opening the door to the possibility that suspects were still under the influence when questioned by the police. Although Palmer and colleagues’ main focus was on witnesses and no in-depth analyses were conducted with this suspect population, that study is to our knowledge the only archival study of intoxicated and drug dependent suspects. However, the study’s focus was on prosecutors and not police. The present study therefore addressed this void by not only examining demographics of intoxicated and drug dependent suspects but also how police conduct interrogations with said suspects, including the dynamic interrogator-suspect interaction and the outcomes of the interrogations.

To evaluate the quality of interrogations there is a need to establish what is currently considered best scientific practice. In the investigative interviewing literature, a distinction has been made between information-gathering and accusatorial interrogation methods. The key difference is the overall goal of the interrogation, with the former focused on gathering accurate information and the latter on eliciting a confession, resulting in different interrogation strategies. The accusatorial method has been criticized for being overly confrontational and guilt presumptive whereas information-gathering approaches reduce the risk of false confessions compared to accusatorial approaches, without incurring a substantial drop in true confession rates (see Meissner et al., 2014, for a meta-analysis). Contrary to common beliefs, information-gathering techniques have proven difficult for suspects to counteract and are also cognitively more demanding than accusatorial techniques (Vrij et al., 2006). Overall, legal psychology researchers recommend the use of an information-gathering approach (Vrij et al., 2017). However, the information-gathering/accusatorial distinction is not the only way to classify interrogation approaches.

Kelly et al. (2013) developed the taxonomy of interrogation methods framework, a standardized model for assessment of interrogation techniques intended for researchers as well as practitioners. It included 71 techniques divided into six domains: (1) *Rapport and relationship building*; (2) *Emotion provocation*; (3) *Confrontation/competition*; (4) *Collaboration*; (5) *Context manipulation*; and (6) *Presentation of evidence*. The domains defined within the taxonomy, the authors argued, parsimoniously organize all known interrogation techniques, including those that may not be permissible in different legal contexts (see Izotovas et al., 2021). *Rapport and relationship building* focuses on establishing a working relationship between the two parties by showing empathy, finding common ground and displaying active listening (Vanderhallen et al., 2011; Kelly et al., 2013). The domains *confrontation/competition* and *emotion provocation* are more consistent with accusatorial interrogation methods (Meissner et al., 2014). Tactics connected to *confrontation/competition* focus on asserting authority and control over the suspect. The *emotion provocation* domain contains techniques for manipulating the suspect’s emotions and includes minimization tactics. The *collaboration* domain refers to techniques of negotiating and bargaining with the suspect and *context manipulation* regards environmental and contextual factors (e.g., the size of the room, time of day). The sixth domain, *presentation of evidence*, indicates how and when evidence is presented (Kelly et al., 2013).

In addition to the use of science-based and ethical interrogation techniques and domains, the present study examined an additional cluster of interrogation methods — question types. As suggested by Kelly and Valencia (2021), the types of questions posed by the interrogator are also essential to consider when assessing the nature and quality of interrogations. The use of appropriate question types (e.g., open questions) is considered important for obtaining detailed and accurate information from a suspect (Walsh and Milne, 2008; Oxburgh et al., 2010, 2012; Phillips et al., 2012). The use of inappropriate question types, such as forced choice and leading questions, on the other hand, can result in distorted responses and false confessions (e.g., Milne and Bull, 1999; Gudjonsson, 2003; Oxburgh et al., 2010). This is especially important to consider when dealing with vulnerable suspects, who might be sensitive to leading and guilt presumptive questioning and interrogative pressure (Bowles and Sharman, 2014; Farrugia and Gabbert, 2019, 2020; Kloft et al., 2021). To evaluate the use of question types in the current study, we adapted an established classification system of question types (see Griffiths and Milne, 2006; Oxburgh et al., 2012; Kelly and Valencia, 2021). The question type cluster is divided into two categories: appropriate and inappropriate. The appropriate category contains three question types, *open*, *probing*, and *appropriate closed*. The inappropriate question category contains five question types, *inappropriate closed*, *leading*, *forced choice*, *multiple* and *statements and opinions* from the interrogator. *Appropriate closed* questions address previously discussed topics during the interrogation and are used for clarification, whereas *inappropriate closed* questions address information which the suspect has not yet addressed (Griffiths, 2008).

There has been a fair amount of research as to which interrogation methods are used in practice, and the results differ between countries depending on legislative, cultural and political factors. In a survey comparison across different countries, interrogation practices in the United States and Canada conformed to an accusatorial approach (Miller et al., 2019). Interrogations in Europe, Australia and New Zealand on the other hand, followed a more information-gathering approach. The lack of national policy guidelines in some countries results in different interrogation practices across police stations and investigators within stations. In the case of drug- and alcohol-related crimes, police officers must use their own judgment when deciding when, how and where to interrogate/interview sober, alcohol- or drug-intoxicated suspects, witnesses and victims (see Evans et al., 2009; Crossland et al., 2018; Hagsand et al., 2022a,b; Pettersson et al., 2022). Sweden currently differs from the neighboring Norway, as well as United Kingdom, in that there is no clear national policy for how interrogations should be carried out by the police. However, this is about to change as a new interrogation framework is under development by the Swedish police in collaboration with researchers which is based on the approach used in the United Kingdom (Swedish Police Authority, 2019). As such, the present study addressed the void in empirical knowledge about Swedish interrogation practices, and in the long run, can inform research and national policy guidelines.

Research on Swedish police interrogations is scarce; however, it has yielded some interesting findings. For instance, in a survey of suspects convicted of murder and sexual offenses, suspects were more likely to admit guilt when met with a humane approach (Holmberg and Christianson, 2002). Furthermore, a mixed method study with juvenile suspects of serious crimes found the majority of questions asked by the police to be inappropriate (Winerdal et al., 2018). The interrogating officers also used unwarranted social pressure to gather information and attempted to elicit confessions from the young suspects. A recent study on self-reports from Swedish and Norwegian police also showed that interrogators in child sexual abuse cases used confrontational techniques in their interaction with suspects, especially when the interrogators experienced negative emotions. Anger was displayed by the interrogators as a mean to gain confessions from suspects (Magnusson et al., 2021). Furthermore, a case study that examined interactions between police and one murder suspect identified a pattern where the investigator seemed to switch between showing empathy (with the objective to achieve rapport) and challenging the suspect (with the objective to get information and results) (Jakobsson Öhrn, 2008). A few studies have also examined the prevalence of confirmation bias in Swedish police interrogations and during the investigation process and found that this is a problematic issue (Jakobsson Öhrn and Nyberg, 2009; Lidén, 2018). Also, a new Swedish archival study (Hagsand et al., 2022c), analyzed police interactions with *sober* suspects of serious crimes (e.g., murder, rape) using the taxonomy of interrogation methods framework. The results showed that, in line with previous research from the United States (e.g., Kelly et al., 2016), rapport building techniques were significantly negatively associated with confrontation. Confrontational techniques were commonly used with emotionally manipulative techniques. Rapport building attempts were related to suspect cooperation while confrontational techniques were associated with decreased cooperation.

Taken together, the present research was the first *archival study* to examine the nature and quality of police interrogations in low-stakes crimes with potentially vulnerable suspects, specifically, suspects in drug- and alcohol-related crimes. These types of crimes were selected as the study’s focus, as they were expected to involve a large proportion of suspects who were either intoxicated at the time of interrogation, had ongoing substance use disorder, or both. The taxonomy of interrogation methods framework (Kelly et al., 2013) in conjunction with classification of question types was employed to analyze interrogating officers’ approach.

Specifically, the current study sought to address the following four research questions:

RQ1: What proportion of suspects in the collected sample show signs of intoxication or substance use disorder during the interrogations?

RQ2: What types of (a) interrogation methods and (b) question types are used in law enforcement interrogations of suspects in low-stakes drug- and alcohol-related crimes?

RQ3: Do (a) interrogation methods and (b) question types differ depending on suspect intoxication or substance use disorder?

RQ4: Do (a) suspect cooperation and (b) confessions vary by intoxication or substance use disorder?

## Method

The present study consisted of two archival studies of Swedish police interrogations, testing the taxonomy of interrogation methods framework (Kelly et al., 2013) as one cluster of interrogation domains and question types as another cluster (see Kelly and Valencia, 2021). Study 1 was conceptualized as a smaller pilot study to Study 2. The main method and procedure were the same for both studies, with some adjustments made in Study 2 based on Study 1’s findings. Both studies were approved by the Swedish Ethical Review Board in line with legal regulations in Sweden; Study 1 (Protocol No. 2018-158-18 and No. 2020-00538) and Study 2 (Protocol No. 2020-06960). In line with the approval from the ethical board, a general informed consent was obtained from the Swedish Police Authority giving the authors permission to analyze and present data from the sample of interrogations provided, for the purposes of the present study.

### Material

The sample of interrogations was provided by a large police department in a major Swedish city. As described earlier, legal regulations (Swedish code of juridical procedures, 2017:176, 23 chapter. 21§) states that several factors (e.g., the seriousness of the crime) determine if the police should audio/video record an interrogation and then transcribe it, or if the police only should document the interrogation using notes (hand-written or typed). In less serious crimes where the interrogation is not recorded, the interrogator is obligated to take their own notes of what was said and then read them out loud so that the suspect can approve or disapprove. Hence, to this extent it is likely that suspect-approved interrogation notes correspond well with what actually happened in the interrogation room. Since the focus of the present study was on low-stakes crimes, it is likely that the vast majority of the documentation provided was interrogators’ handwritten or computer-typed notes, although it was not always clear whether the documentation provided to the research team were transcriptions or written notes. As our materials consisted of a mix of notes and transcripts of recordings (although the majority was notes), we will refer to our materials as written police interrogations^1^.

In either case, all documentation provided consisted of detailed accounts – stating exactly what the interrogator and the suspect said. The very detailed notes are most likely evidence that the vast majority of the material in the present study was so called contemporaneous notes – i.e., notes taken by the police while conducting the interrogation or in close temporal proximity to that, suggesting that these notes were an accurate reflection of what was actually said. As an example, here follows a quote of an interaction between one police (P) and one suspect (S) in an interrogation belonging to the present sample: P: *“Do you understand the charges?”* S: “Yes.” P: *“How do you respond to these charges?”* S: “I confess” P: “*What is it that we have found on you?”* S: “Cocaine” P: “*Where did you buy it?”* S: “I don’t remember.” P: *“When did you buy it?”* S: “On Friday. It was downtown, I was just out and bought it of someone. I do not know who it was.” Previous published studies in legal psychology have used contemporaneous notes from police interviews with real-world witnesses (Orbach et al., 2012), and from interviews with participants as witnesses (Powell et al., 2011), and scientists have also examined how contemporaneous notes affect jurors’ decision making (Deck and Paterson, 2021). The use of contemporaneous notes in previous research argues that this is a solid scientific method. It is also important to highlight that the notes analyzed in the present study were not summary notes. In cases where only a summary (e.g., “The suspect was asked about the crime, he explained he was at the crime scene, and he confessed”) of what was said was obtained from the Swedish Police Authority, these interrogation summaries were excluded from the final sample (see more details below).

### Sample

#### Study 1

The pilot study included written interrogations (*N* = 39) conducted between 2016 and 2017, with suspects in either alcohol-related (i.e., DUIs) or drug-related (i.e., drug use and/or possession) offenses. The Swedish Police Authority issued a non-disclosure agreement and granted access to the raw material (i.e., written interrogations including personal information with names etc.) only to the first author, who in turn de-identified all written interrogations (e.g., removing names, addresses and social security numbers) for coding purposes. Initially, 48 police interrogations were collected. However, nine interrogations were excluded because they were either non-detailed summaries of the interrogations (*n* = 3), too short to analyze as in the case of a dialogue containing only two sequences between the interrogator and suspect (*n* = 1) and reviews of confiscated items (*n* = 1). Also, repeated interrogations of the same suspect (*n* = 3) which might interfere with the data-analysis, and an interrogation that was a corrupted file that could not be opened (*n* = 1) were also excluded. To avoid contamination from previous interrogations only the *first* interrogation in each case with repeated interrogations was included. The final sample consisted of 39 police interrogations with 38 male suspects and one female suspect. Suspects’ ages at the time of the interrogation ranged from 16 to 65 (*M* = 35.28, *SD* = 13.10) (see Table 2 for sample characteristics). Interrogation length ranged from 7 to 107 min, with a mean of 26.36 min (*SD* = 21.82).

**TABLE 1:** Distribution of crime classifications within the sample in Study 1 and Study 2.

Crime classification	Study 1 (*N* = 39)	Study 2 (*N* = 97)
	
	*n* (%)	*n* (%)
Minor drug offenses (drug use or possession)^a^	19 (48.7)	90 (92.8)
Unlawful driving/DUI	24 (61.5)	10 (10.3)
Reckless driving	3 (7.7)	4 (4.1)
Serious drug offenses/possession of drugs for distribution	2 (5.1)	1 (1.0)
Illegal carrying/unlawful possession of a weapon	2 (5.1)	11 (11.3)
Theft/dealing in stolen goods	1 (2.6)	2 (2.1)
Damage of property	0 (0.0)	2 (2.1)
Threat to a public servant/unlawful threat	1 (2.6)	12 (12.4)
Assaulting an officer/public servant	0 (0.0)	16 (16.5)
Violent resistance	2 (5.1)	9 (9.3)
Obstruction of justice	0 (0.0)	2 (2.1)
Harassment	0 (0.0)	1 (1.0)
Sexual harassment	1 (2.6)	0 (0.0)
Money laundering	0 (0.0)	1 (1.0)
Assault/attempted assault	1 (2.6)	2 (2.1)
Aggravated assault/attempted manslaughter	1 (2.6)	4 (4.1)
Rape/attempted rape	0 (0.0)	3 (3.1)
Murder/attempted murder	0 (0.0)	2 (2.1)

^a^All cases but not all interrogations involved minor drug charges.

**TABLE 2:** Characteristics of the interrogation sample in Study 1 and Study 2.

	Study 1	Study 2
Total number of interrogations	39	97
**Gender suspect**		
Male	38	82
Female	1	15
**Gender interrogator**		
Male	28	43
Female	11	54
**Other information**		
Unique interrogators^1^	37	71
Informed right to attorney	39	97
Attorney present	2	19
Interrogation witness present	5	3
Interpreter present	5	10
**Time of interrogation**		
Morning (5.00 am – 10.00 am)	7	13
Midday (10.00 am – 1.00 pm)	5	11
Afternoon (1.00 pm – 6.00 pm)	6	28
Evening (6.00 pm – 11.00 pm)	6	23
Night (11.00 pm – 5.00 am)	14	22
**Place of interrogation**		
Police station	26	66
On the scene	4	14
Holding cell	3	0
Police car	2	7
Other location	4	5
Not stated	0	5
**Interrogation outcome**		
Confession (full or partial)	27	68
Denial	12	29
**Conviction drug- and/or alcohol-related crime**		
Convicted drug- and/or alcohol-related crime	25	51
Acquitted drug- and/or alcohol-related crime	2	6
**Conviction other crimes**		
Convicted other crimes	14	38
Acquitted other crimes	2	8

^1^“Unique interrogators” refer to each individual police interrogator. As an example, in Study 2 there were a total of 97 interrogations, but some were conducted by the same interrogator. Hence, there were 71 unique police interrogators in that sample.

#### Study 2

The main study included written interrogations (*N* = 97) conducted between 2019 and 2020. All suspects were interrogated about drug-related offenses (although some cases also addressed alcohol-related crimes). In 54.0% (*n* = 52) of the interrogations, suspects were questioned regarding additional crimes, ranging from petty theft to murder (see Table 1 for crime classifications). In Study 2, both the first author and two coders were included in the non-disclosure agreement with the Swedish Police Authority. The initial sample consisted of 183 interrogations. Some interrogations (*n* = 54) were excluded, being either non-detailed summaries of the interrogations (*n* = 29) or being too brief (*n* = 25) to analyze (less than six sequences of dialog between police and suspect). Interrogations with minors (<18 years), were also excluded (*n* = 8). Repeated interrogations (*n* = 24) with the same suspect were also excluded, as they could interfere with the analysis. To avoid contamination from previous interrogations only the *first* interrogation in each case with repeated interrogations was included. In instances where the first interrogation had been excluded for being too short or the first interrogation was aborted (e.g., the police tried to conduct the interrogation, but the suspect did not want to engage) the second interrogation was used instead. In these cases, it was deemed that the initial interrogation attempt would not influence the “real” (second) interrogation and could thus be included in the analysis. The final sample therefore consisted of 97 interrogations of unique suspects, with the exception of one suspect who reappeared months later in a different criminal case. The final sample consisted of 82 male suspects and 15 female suspects with ages at the time of the interrogation ranging from 18 to 65 (*M* = 32.67, *SD* = 10.85). In one case the exact age could not be determined. See Table 2 for sample characteristics. The mean interrogation length was 27.96 min (*SD* = 30.11), ranging from 4 to 158 min.

### Procedure

The material was coded and analyzed using the method outlined by Kelly and Valencia (2021) with integrated elements from the taxonomy of interrogation methods framework (Kelly et al., 2013, 2016). In line with Kelly and Valencia (2021), the interrogating officers’ behaviors were analyzed in two clusters – interrogation domains and question types. Beyond this, a novel coding template was developed to code if suspects showed signs of intoxication or addiction. The scorers in Study 1 were advanced students in legal psychology. In Study 2, the scorers, with master’s degrees in (legal) psychology, were employed in the current project and were very familiar with the coding procedures, including the new coding scheme for intoxication and substance abuse.

The written interrogations were coded in sequences, where one sequence was defined as a question or statement from the interrogator, and a response or non-response from the suspect in reply (“*Do you understand the charges*?” and “*Yes/No*” or silence from the suspect). Similar procedures of sequencing interrogations or interviews can be found in other studies (e.g., Schreiber Compo et al., 2012; Hagsand et al., 2022c). In Study 1, the 39 interrogations contained 561 sequences in total (*M* = 14.36, *SD* = 11.29, range: 5–59). In Study 2, the total number of sequences was 1965 (*M* = 20.26, *SD* = 18.04, range: 6–136). In line with previous studies (e.g., Kelly et al., 2016; Kelly and Valencia, 2021; Hagsand et al., 2022c), interrogations were divided into three temporal blocks – beginning, middle, and end, using the number of sequences in the interrogation as the numerator when dividing the interrogation into thirds. The sample in Study 1 consisted of 117 (3 × 39) blocks and the sample in Study 2 consisted of 291 (3 × 97) blocks. Furthermore, in Study 1, question types were only coded in the absence of interrogation techniques, but in Study 2 both question types and interrogation techniques were coded in the same sequence. Accounting for this potential overlap gave a more comprehensive and realistic reflection of the interaction, therefore improving the method in Study 2. This is also congruent with the method used by Kelly and Valencia (2021).

#### Interrogation techniques and domain emphasis

The taxonomy of interrogation methods framework (Kelly et al., 2016) was applied to code the use of interrogation techniques in each sequence. In the original taxonomy, interrogation techniques were divided into six domains, *rapport and relationship building*; *context manipulation*; *emotion provocation*; *confrontation and competition*; *collaboration;* and *presentation of evidence.* In the current studies, the domain *context manipulation* was excluded since it was difficult to determine contextual details from written interrogations. The *collaboration* domain was coded but excluded before the analysis in both studies because it was only found once in Study 1 and was not found at all in Study 2. The decision to exclude these domains is congruent with previous studies examining the domains (e.g., Izotovas et al., 2021; Kelly and Valencia, 2021). In the present research, the coding templates^2^ are available on the Open Science Framework; OSF, for Study 1 and Study 2 consisting of 60+ techniques belonging to the different domains.

Based on the coding process of Study 1, changes to the coding template were made for Study 2. In the *rapport and relationship building* domain, the technique *ask for free account* (i.e., free recall), which is common in the practice of investigative interviewing, was added as it was not part of the original classification of appropriate or inappropriate question types, or belonging to any of the domains. As the purpose behind asking for a free account is more than just asking a direct question, it was added to the domain *rapport and relationship building* instead of to the question types classifications. Because the technique could be part of establishing an open and tolerant atmosphere in the initial phase of an interrogation, it was deemed suitable for the *rapport and relationship building* domain. In the *emotion provocation* domain, the technique *taunting/provoking* was added. In this technique, the interrogator identifies topics, utterances or behaviors that seem to provoke the suspect and uses them in attempts to incite or enhance feelings of anger and/or frustration. In the *confrontation/competition* domain, the technique *prompt speculation* was added. In this technique the interrogator asks or demands that the suspect speculate about what might have happened, often accompanied with the explicit notion that if the suspect themselves did not do it, they must be able to provide a plausible alternative explanation. Not providing an alternative explanation is then often interpreted by the interrogator as a sign of guilt. Furthermore, for Study 2, in the *presentation of evidence* domain, some techniques were removed since they were either illegal (*bluff the suspect about supposed evidence* and *confront the suspect with supposed evidence*) or highly unlikely to be used in a Swedish context (*use polygraph or other physiological measures*). Although not included in the coding manual, the coders were aware of these techniques during the coding process and did not identify any such techniques while coding, strengthening the decision to exclude them. In the pilot study, Study 1, these techniques were included in the coding template, but they were not identified during the coding.

Each sequence was coded for the presence (1) or absence (0) of interrogation techniques. Although most sequences included only a single identifiable technique, the coding did allow for those instances when multiple techniques from both the same and from different domains could be coded in a single sequence. The frequency of techniques (coded as 1 or 0) within each domain were summed within sequence in order to obtain domain scores for analysis. To obtain measures for the block level interrater reliability analyses, domain emphasis was then coded on a three-point scale (0 = none, 1 = moderately, 2 = greater/exclusively) for each temporal block. The aim of the scale was to emphasize which domains were used in the various sections of the interrogation, and to what degree (Kelly et al., 2016; Kelly and Valencia, 2021).

In Study 1, domain emphasis was calculated based on an equation^3^ (see Hagsand et al., 2022c, for more details) which was used as a cut off point for coding a 1 or 2 for domain emphasis in the respective domain and temporal block. However, in Study 2, the authors examined whether alternative methods for obtaining domain emphasis resulted in similar outcomes. We found overwhelming congruity in the outcome between the equation used in Study 1 and the method used by Kelly et al. (2016) and Kelly and Valencia (2021). Therefore, in Study 2, coders subjectively determined domain emphasis based on the frequencies of coded techniques belonging to each domain, as previous studies by Kelly and colleagues have done.

The interrater reliability coding for both studies was performed on approximately 20% of the material and calculated on block level domain emphasis scores and then averaged for the whole interrogation. Krippendorff’s alpha, α (KALPHA) was considered sufficient at a level of α ≥ 0.60 for this complex coding^4^. Due to the complexity of the coding and that KALPHA was misleading in some cases (i.e., adjustments penalizing coders for the large number of zeros coded in the absence of interrogation techniques), percentage agreement was also calculated. For Study 1, interrater reliability was calculated for the domain scores on the block level, for a randomly selected nine interrogations (23.1% of the total sample). Acceptable reliability was achieved (*rapport and relationship building* α = 1.00, 100%; *emotion provocation* α = 0.00, 96.3%; *confrontation and competition* α = −0.02, 92.6%; *collaboration* α = 1.00, 100%, and *presentation of evidence* α = 0.78, 96.3%). For Study 2, interrater reliability was calculated on 27 randomly selected interrogations (27.8% of the total sample). An acceptable interrater reliability level for each of the domains was met (*rapport and relationship building* α = 0.75, 92.6%; *emotion provocation* α = 0.66, 93.8%; *confrontation and competition* α = 0.90, 93.8%; *collaboration* 100% and *presentation of evidence* α = 0.88, 91.4%).

#### Question types

The question types used in for example Kelly and Valencia’s (2021) study are categorized as appropriate (open, probing, appropriate closed) and inappropriate (forced choice, leading, multiple questions, statements, inappropriate closed) as per the coding template (see text footnote 2) (both Study 1 and 2). The presence of each question type was coded on the sequence level, similar to the coding of interrogation techniques. For the present study, a new separate category called “routine information/neutral statements” was also added to the coding. This category consisted of sequences that did not fit into any existing categories of appropriate and inappropriate question types. Namely, routine information phrased as dialogue or utterances (i.e., “That concludes this interrogation. The time is 14.03”) not in the form of a question, opinion, or an interrogation technique. For Study 2, the “routine information/neutral statements” category could have an overlap with the coding of either techniques in the domains and/or with question types (in the case were several different things happened during the same sequence).

A single measure, the Appropriate Question Differential (AQD), developed by Kelly and Valencia (2021), was then used to determine the degree of appropriate and inappropriate questions for the whole interrogation. AQD was measured on a scale ranging from −1 to +1 (−1 = only inappropriate questions asked, 0 = no questions asked at all or equally many appropriate as inappropriate questions, +1 = only appropriate questions asked). AQD was calculated with the equation (Appropriate questions – Inappropriate questions)/(Appropriate questions + Inappropriate questions).

For Study 1, interrater reliability (calculated on sequence level) for appropriate question types was α = 0.66 (93.9%), α = 0.66 (93.9%) for inappropriate question types and α = 0.88 (93.9%) for total question types. For Study 2, KALPHA agreement (calculated on sequence level) for appropriate question types were α = 0.59 (87.5%), α = 0.66 (95.9%) for inappropriate question types and α = 0.83 (91.7%) for total question types.

#### Suspect cooperation

The coding instructions (available on OSF) (see text footnote 2) outlined in Kelly et al. (2016) were used for coding of suspect cooperation. The suspects’ response in *each sequence* was coded on the dimensions *cooperation* (0–2) and *resistance* (0–2) and intended to capture the suspects’ statements regardless of the self-incriminating character of the information or its reliability. The scale of 0–2 points was defined as 0 = not present, 1 = somewhat present, and 2 = strongly present (see Kelly et al., 2016). The suspect’s *cooperation* included providing (a) non-incriminating information (whether related or not to the offense), (b) self-incriminating information (admissions or confessions), or (c) alibis or reasons that the suspect could not have committed the offense. The category *resistance* included (a) denials of all allegations, (b) statements of bad memory or lack of knowledge, (c) silence or non-answers, (d) repudiation of previous admissions or confessions, and (e) references to, or calling upon, the use a defender and other rights. The scores were then converted into a single 5-point measure (1 = strong resistance; 2 = weak resistance; 3 = neutral; 4 = weak cooperation; 5 = strong cooperation). Neutral (3) represents values where there the suspect was both cooperative and resistant in the same sequence, canceling each other out. The described 5-point scale constituted the final outcome variable for suspect cooperation.

For Study 1, interrater reliability (calculated on sequence level) for the suspect cooperation coding achieved a KALPHA of α = 0.67 (82.1%), and for Study 2 a KALPHA of α = 0.67 (46.1%).

#### Suspects’ confessions versus denials

In order to statistically compare interrogations in which the suspect confessed versus denied, a categorization of the interrogations was made. The categorization of suspect confession (at the interrogation level) was based on Kelly et al. (2016). If suspects confessed to at least one of the crimes they were accused of, the interrogation was coded as one (1) (e.g., “*I confess*”), while straight-forward denials (e.g., “*I did not do it*”) or no comments/withdrawal of confession were coded as two (2). In cases where it was not possible to determine from the written interrogations if the suspect confessed or denied, this was coded as a three (3). Whether the suspect confessed or denied was information that was explicitly stated in the interrogations since the interrogator always asked how the suspect responded to the charges (e.g., P: “*Do you confess or deny?”* S: “I confess”). Thus, there was no need for a specific coding framework or interrater reliability coding, and this is also in line with Kelly et al. (2016) who did not calculate interrater reliability on this variable.

#### Coding of signs of intoxication during interrogations

An additional coding scheme was developed to enable analysis of which suspects were intoxicated with drugs and/or alcohol *during* the interrogations (i.e., which may or may not correspond with intoxication at the time of the crime). The coders were instructed to assess both what was said during the interrogation, and the context (e.g., time of day, place of interrogation).

##### Study 1

Based on a coding manual (see text footnote 2) for intoxication, two coders categorized each suspect in the interrogation into one of five categories: (1) alcohol-intoxicated, (2) drug-intoxicated, (3) under the influence of both alcohol-and drugs, (4) sober, and (5) not possible to determine intoxication based on the written interrogations. Interrater reliability was calculated based on the coding on the interrogator level on nine (23.1% of total sample) of the interrogations and an acceptable agreement of α = 0.80 (88.9%) was achieved.

##### Study 2

For Study 2, the coding template (see text footnote 2) for intoxication was refined such that hangover and withdrawal were coded in addition to intoxication. Both written interrogations from the Swedish Police Authority and court hearing documents including the verdicts from the district courts (i.e., court hearing documents announcing the verdict and summarizing the evidence and circumstances leading up to the verdict and penalty – also including explicit information about intoxication and/or substance use) were used in the coding. Coders were instructed to categorize each suspect into one of the three categories: (1) Evidence of intoxication, (2) Evidence of hangover/withdrawal, or (3) No evidence of intoxication or hangover/withdrawal during interrogation. To be able to categorize intoxication and hangover/withdrawal, the coders looked for specific criteria (e.g., suspects statements of intoxication and/or consumption, contextual factors and objective measures of intoxication) where at least one had to be met. To further assist in the coding, a comprehensive coding template (see text footnote 2) of substances with information about their effects, duration time and possible interaction effects with other drugs was developed for the coders. The coding template was used to assure that the two coders had access to the same information. Based on the available written material, coders determined whether there was evidence of intoxication or not, that is, whether a suspect showed *signs* of intoxication or not, underscoring the subjective nature of the coding possible in this study (as opposed to for example breathalyzer tests or blood samples). Interrater reliability was calculated based on the coding (on the interrogation level) on 29 (29.9% of total sample) of the interrogations and an acceptable agreement of α = 0.81 (79.3%) was reached.

#### Coding of signs of substance use disorder

As a further development in Study 2, a coding of signs of suspect use disorder abuse was conducted because this factor in itself might alter the interrogation even when the suspect is not currently intoxicated (e.g., Davison and Gossop, 1996; Sigurdsson and Gudjonsson, 1996; Santtila et al., 1999; Gudjonsson et al., 2004). It was often stated explicitly during the interrogations that the suspect had an ongoing drug problem but for less obvious cases, an extensive coding framework needed to be developed. Since no such coding to our knowledge had been done, a new coding manual (see text footnote 2) for signs of substance use disorder was developed by the research team. The coding template was based on the Diagnostic and Statistical Manual of Mental Disorders (DSM-V) manual and the 11 criteria for *substance use disorder* (American Psychiatric Association, 2013). A development was made in DSM-V, so that substance use disorder is a new category that consists of the former two DSM-IV categories, that is, substance abuse and substance dependence. The new categorization was measured on a single continuum from mild to server substance use disorder. With this said, the coding in the present study was complex and many criteria in the DSM-V are based on the subjective experience of substance use disorder. The intention was not to clinically diagnose the suspects with substance use disorder, but rather the DSM-V criteria were used as an indication of whether the suspect appeared to have a substance abuse problem or not. We therefore oftentimes state that a suspect showed *signs* of substance use disorder or not in the present study, in an attempt to underscore the subjective nature of the coding performed instead of a proper clinical assessment.

The coding template categorized the suspects into two different categories: (1) Evidence of substance use disorder; (2) No evidence of substance use disorder. To be able to code the prevalence of substance abuse three criteria were followed, where at least one had to be met for substance abuse to be coded, (1) The written interrogations and/or the court hearing documents including the verdicts contained explicit information about the suspect suffering from substance use disorder; (2) The written interrogations and/or the court hearing documents including the verdicts contained information about the suspect currently being under treatment for substance use disorder; and (3) *Two or more DSM-V criteria* for substance use disorder were met.

Interrater reliability was calculated based on codings (on the interrogation level) on 29 (29.9% of the total sample) of the interrogations and an acceptable interrater agreement of α = 0.56 (82.8%) was met. In Study 2, we also explored which substances were used by suspects and this is reported in the result section. This information was based on the written interrogations and court hearing documents. For that coding, coders achieved an agreement of 100%. No additional coding frame was used for the examination of which substances were used by the suspects. This information was readily available both in the interrogations and the court documents. When judging which substances suspects were addicted to and/or under the influence of during interrogation, we consulted the results from the previous coding of intoxication and substance abuse and the list of substances, as described above. The interrater reliability agreement for that categorization was acceptable at a level of α = 0.63 (86.2%).

#### Units of observation and statistical analysis

To be clear, we observed the interrogation clusters, suspect responses, and suspect intoxication and substance use disorders at two different levels that have implications for the statistical analyses that we conducted and that are reported in the results section. First, the detailed sequence level coding included the interrogation techniques and domains, specific question types, and suspect cooperation in order to address Research Question 2. Next, these variables were averaged for an interrogation level measurement. Whether the suspect confessed and whether there were signs of suspect intoxication and substance use disorder were also measured at this level. Research Questions 1, 3, and 4 were addressed at the interrogation level.

#### Statistical power

Study 1 was intended as a pilot study due to the small sample size, hence no power calculation was performed. For the main study (Study 2), power calculations using G*Power (Faul et al., 2007) showed acceptable statistical power for these analyses. For example, the *t*-tests examining differences in suspect cooperation between groups (*t*-test 1: signs of intoxication vs. not, *t*-test 2: signs of substance abuse disorder vs. not) showed a 67% and 68% chance, respectively, to detect medium effect sizes (*d* = 0.50), and +97% power to detect large effect sizes. The chi-square tests, examining differences in confession rates between groups, displayed an 84% chance to detect medium effect sizes (*w* = 0.30) and a 99% chance to detect large effect sizes (*w* = 0.50).

## Results

### RQ1: Proportion of suspects showing signs of intoxication or substance use disorder

The coding of intoxication in both samples indicated that the majority of the suspects were either intoxicated, hungover or experiencing withdrawal. In Study 1 (*N* = 39), 28.2% (*n* = 11) of the suspects were sober, 28.2% (*n* = 11) were under the influence of alcohol, 15.4% (*n* = 6) were under the influence of drugs, and 10.26% were under the influence of both alcohol and drugs. In 18.0% (*n* = 7) of cases, it was not possible to determine intoxication level. In Study 2 (*N* = 97), 22.7% (*n* = 22) of the suspects were intoxicated, 35.1% (*n* = 34) were hungover or withdrawing, and in the remaining 42.3% (*n* = 41), signs of intoxication could not be determined, and the suspect was therefore coded as sober. In Study 2, an additional code was utilized to explore which substances were used by the suspects. It revealed that most suspects were under the influence of alcohol during the interrogation (*n* = 22), followed by amphetamines (*n* = 20), cannabis (*n* = 15), and benzodiazepines (*n* = 15). Of the 56 suspects who were intoxicated during the interrogation, 24 were under the influence of multiple substances (see Table 3, for a detailed overview). The coding of signs of substance use disorder, conducted in Study 2 only, suggested that 56.7% (*n* = 55) suspects suffered from this issue. A coding of which substances suspects were habitually using/dependent on indicated that amphetamines (*n* = 24), benzodiazepines (*n* = 19), and cannabis (*n* = 19) were most common. Of the 55 suspects who were showing signs to suffer from substance use disorders, as many as 40% (*n* = 22) were habitually using multiple substances (see Table 3, for a detailed overview).

**TABLE 3:** Table of substances and prevalence of intoxication and substance abuse in Study 2 (*N* = 97).

Substance	Number of suspects using the substance	Suspects under influence of the substance during interrogation	Number of suspects addicted to the substance
Alcohol	30	22	10
Cannabis	37	15	19
Benzodiazepines	38	15	19
Opioids	16	3	8
Amphetamines	35	20	24
Cocaine	15	12	5
Ecstasy/MDMA	3	0	1
GHB	3	2	1
Other	11	2	2

### RQ2a: Frequencies of interrogation techniques and domains

For the total number of 561 sequences over 39 interrogations in Study 1, only 8.2% of sequences contained any of the interrogation techniques specified within the five domains of the taxonomy (Kelly and Valencia, 2021). Of the interrogation domains used, the most common was *presentation of evidence* (50.0%) and *confrontation/competition* (14.6%). In total, 18 techniques were found in the intoxicated group and 16 were found in the sober group. Due to the low frequency of coded techniques, no statistical analyses were conducted for Study 1. In Study 2, only 12.2% of the 1965 sequences contained interrogation techniques belonging to the domains. *Presentation of evidence* was the most frequently used domain with 50.4% of all coded techniques falling under this domain. It was followed by *rapport and relationship building* (28.3%), *confrontation/competition* (21.7%) and *emotion provocation* (6.7%). For descriptive information about the different domains, see Table 4.

**TABLE 4:** Descriptive information for question categories, specific question types, AQD and domains in Study 2 (*N* = 97).

	Mean (SD)	Min	Max
**Question type**			
**Appropriate**	**17.09 (15.71)**	**3**	**119**
Open	1.30 (2.71)	0	21
Probing	9.30 (8.97)	0	62
Appropriate closed	6.48 (5.33)	1	36
**Inappropriate**	**1.15 (1.47)**	**0**	**6**
Inappropriate closed	0.37 (0.79)	0	5
Leading	0.11 (0.43)	0	3
Multiple	0.18 (0.54)	0	4
Statement	0.02 (0.14)	0	1
Forced choice	0.46 (0.83)	0	3
**Appropriate Question Differential (AQD)**	**0.85 (0.20)**	**0**	**1**
**Domains**			
Rapport and relationship-building	0.19 (0.46)	0	2
Emotion provocation	0.05 (0.24)	0	2
Confrontation/competition	0.17 (0.46)	0	2
Presentation of evidence	0.30 (0.62)	0	2

Question types were on the interrogation level. The AQD scale ranged from −1 to +1 on the interrogation level. The domain emphasis scale ranged from 0 to 2 on the interrogation level.

To further examine the nature of the interrogations in Study 2, we identified which specific techniques, from the respective domains were most prevalent. The most frequently coded technique in the *rapport and relationship* building domain was *asking for free account*. *Employing active listening* techniques was also relatively common. In the domain *emotion provocation*, a few instances of *appealing to the suspects self-interest* or *conscience*, as well as the minimizing technique of *offering rationalization*, were found. In the confrontation domain, the majority of the techniques were *prompt speculation* and *ask the same question repeatedly*. For a summary of all techniques coded, see Table 5.

**TABLE 5:** Frequencies of separate techniques in Study 2 (*N* = 97).

	Times coded
**Rapport and relationship building**	
Identify and meet basic needs	1
Let the suspect play the role of the teacher	1
Show concern for the suspect’s situation	9
Use similar language as suspect	4
Employ active listening techniques	16
Straightforward honesty	4
Depersonalize the situation	1
Non-crime-related conversation	1
Ask for free account	31
Use humor to defuse tension	1
Total	69
**Emotion provocation**	
Appeal to the suspect’s self-interest	4
Appeal to the suspect’s conscience	5
Offer rationalizations	5
Total	14
**Confrontation/competition**	
Obscure the fate of the suspect	1
Ask the same question repeatedly	19
Do not allow denials	1
Disparage or dismiss the information provided by the suspect	1
Use the suspect’s own words in a manner that misconstrues or alters the intent	2
Prompt speculation	28
Total	52
**Presentation of evidence**	
Confront suspect with evidence of their involvement	49
Identify contradictions within the story	21
We know all	2
Present statements from witnesses or co-suspects	34
Use audio/visual aids	15
Refer to the suspect’s criminal history	1
Total	122
**Summed total**	**257**

### RQ2b: Frequencies of question types

In Study 1, question types were coded in 92.0% of the sequences (out of which 83.7% of the questions were classified as appropriate and 12.0% as inappropriate), and the rest of the sequences (8.0%) were coded as routine information/neutral statements not belonging to either category (e.g., “you are suspected of violent resistance,” “that concludes this interrogation, the time is 2 PM”). The overall AQD mean score for the whole sample was 0.75 (*SD* = 0.24). In Study 2, question types were present in 90.1% of the sequences (out of which 93.7% of the questions were of the appropriate type and 6.3% of the inappropriate type), and 4.4% of the sequences were counted as routine information/neutral statements (but note that Study 2 allowed overlap which made it possible to code both interrogation techniques, question types and routine information/neutral statements in the same sequence). With respect to the proportion of appropriate to inappropriate questions, the overall AQD mean score was 0.85 (*SD* = 0.20). For descriptive information about the different question types, as well as AQD scores, see Table 4.

### RQ3a: Use of interrogation methods depending on signs of suspects’ intoxication or substance use disorder

**FIGURE 1:**
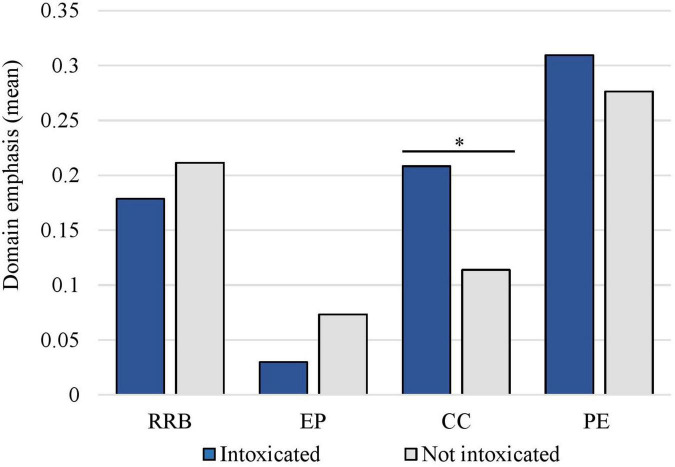
The interrogators’ use of domains with suspects showing signs of intoxication versus not. RRB, rapport and relationship building; EP, emotion provocation; CC, confrontation/competition; PE, presentation of evidence. The domain emphasis scale ranged from 0 to 2 scores. *Significant difference between groups (*p* = 0.042, η^2^ = 0.02).

Due to the small sample size in Study 1, no statistical comparisons between groups were made. For Study 2, to examine differences in domain emphasis scores depending on ongoing suspect intoxication during the interrogation, a non-parametric Kruskal Wallis test was conducted with the domain scores for the four domains (*rapport and relationship building*, *emotion provocation*, *confrontation/competition*, and *evidence presentation*) as dependent variables. The analysis was conducted at the interrogation level (i.e., the whole interrogation) because the independent variable, intoxication (yes/no), concerns the whole interrogation and does not vary during each interrogation. The results revealed that significantly more interrogation techniques from the *confrontation/competition* domain were used with intoxicated suspects than sober ones [*H* (1) = 4.15, *p* = 0.042, η^2^ = 0.02]. No significant differences were detected in the domains *rapport and relationship building* [*H* (1) = 0.14, *p* = 0.705, η^2^ = 0.01], *emotion provocation* [*H* (1) = 2.25, *p* = 0.133, η^2^ = 0.01] and *presentation of evidence* [*H* (1) = 0.19, *p* = 0.661, η^2^ = 0.001]. These results can be further examined in Figure 1, displaying differences in domain emphasis between the groups.

**FIGURE 2:**
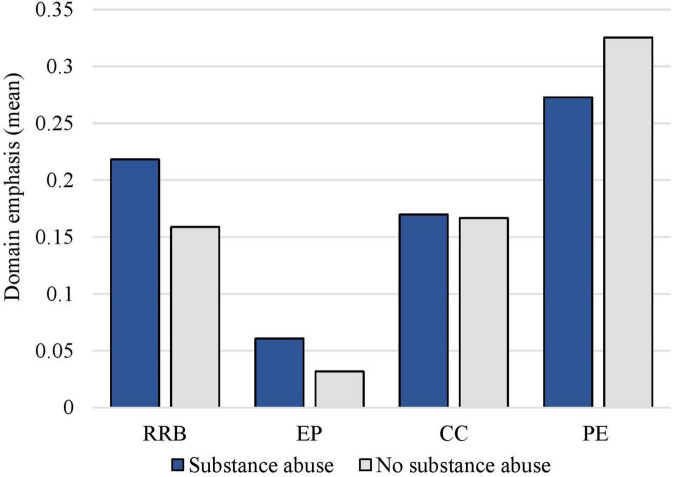
The interrogators’ use of domains with suspects showing signs of substance use disorder versus not. RRB, rapport and relationship building; EP, emotion provocation; CC, confrontation/competition; PE, presentation of evidence. The domain emphasis scale ranged from 0 to 2 scores.

A second Kruskal Wallis test was conducted to examine possible differences in domain emphasis scores depending on whether there were signs of suspects suffering from substance use disorder or not. The results showed no significant difference between the two groups in the domains, *rapport and relationship building* [*H* (1) = 0.56, *p* = 0.455, η^2^ = 0.004], *emotion provocation* [*H* (1) = 0.30, *p* = 0.587, η^2^ = 0.01], *confrontation/competition* [*H* (1) = 0.52, *p* = 0.473, η^2^ = 0.001], and *presentation of evidence* [*H* (1) = 0.02, *p* = 0.883, η^2^ = 0.002]. Differences in domain emphasis between the groups are displayed in Figure 2.

### RQ3b: Use of question types depending on signs of suspects’ intoxication or substance use disorder

Due to the small sample in Study 1, no statistical comparisons between groups were made. In Study 2, differences in AQD scores (proportion of appropriate and inappropriate question types) were examined through two separate *t*-tests, conducted at the interrogation level, with the independent variables of intoxication (yes, *n* = 56/no, *n* = 41) and substance abuse (yes, *n* = 55/no, *n* = 42) and AQD scores as dependent variable. The first *t*-test revealed no significant difference between intoxicated (*M* = 0.83, *SD* = 0.25) and sober (*M* = 0.88, *SD* = 0.12) suspects [*t*(85) = −1.41, *p* = 0.203, *d* = 0.20]. In the second *t*-test, no significant difference was detected between suspects showing signs of substance use disorder (*M* = 0.86, *SD* = 0.21) or not (*M* = 0.83, *SD* = 0.21), [*t*(95) = 0.74, *p* = 0.459, *d* = 0.20] on AQD.

### RQ4a: Differences in suspect cooperation depending on signs of intoxication or substance use disorder

In Study 2, two separate *t*-tests were conducted on the entire interrogation level to examine differences in suspect cooperation (scale 1–5) between (1) suspects showing signs of intoxication versus sober suspects, and (2) suspects showing signs of substance use disorder or not. The first *t*-test found no significant difference in suspect cooperation between intoxicated (*M* = 3.67, *SD* = 0.78) and sober (*M* = 3.72, *SD* = 0.64) suspects [*t*(95) = −0.33, *p* = 0.74, *d* = 0.73]. The second *t*-test, however, revealed that suspects showing signs of substance use disorder (*M* = 3.82, *SD* = 0.67) were significantly more cooperative than suspects without substance issues (*M* = 3.52, *SD* = 0.77), [*t* (95) = 1.99, *p* = 0.049, *d* = 0.71].

### RQ4b: Differences in suspect confession rates depending on signs of intoxication or substance use disorder

Due to the small sample in Study 1, no statistical comparison between groups was made. For Study 2, to examine differences in suspect confession rates, two separate chi-square tests were carried out. The first test, focusing on suspects showing signs of intoxication versus not and confession rates, was non-significant [χ^2^(1, *N* = 97) = 0.61, *p* = 0.503, φ = 0.08]. See Table 6 for more details. The second test showed a significant difference between suspect showing signs of substance use disorder versus not and confession rates [χ^2^(1, *N* = 97) = 5.94, *p* = 0.024, φ = 0.25], where suspects with substance use disorder gave significantly more confessions than the group of suspects without these issues. See Table 7 for more details.

**TABLE 6:** Interrogation outcomes as a function of suspects’ intoxication status in Study 2 (*N* = 97).

		Confessions (%)	Denials (%)	Total
**Signs of intoxication**	Yes	41 (73.2)	15 (26.8)	56
	No	27 (65.9)	14 (34.2)	41
**Total**		68 (70.1)	29 (29.9)	97

**TABLE 7:** Interrogation outcomes as a function of suspects’ substance use disorder status in Study 2 (*N* = 97).

		Confessions (%)	Denials (%)	Total
**Signs of substance use disorder**	Yes	44 (80.0) *	11 (20.0)	55
	No	24 (57.1) *	18 (42.9)	42
**Total**		68 (70.1)	29 (29.9)	97

*Significant difference between groups (*p* = 0.024, φ = 0.25).

## Discussion

### Summary

An important aim of the current research was to assess the nature of Swedish police interrogations in low-stakes crimes related to alcohol and drugs, and the outcome of such interrogations. Our archival sample of interrogations contained a large number of suspects who showed signs of being intoxicated during the interrogation or who appeared to suffer from substance use disorders, which corresponds well with national (Hagsand et al., 2022a) and international (e.g., Evans et al., 2009; Monds et al., 2021) police survey data. Most importantly, our data suggest that suspects who show signs of substance use disorder may be both more likely to be cooperative during an interrogation and more likely to confess compared to suspects without a substance use disorder, at least in the context of low-stakes crimes related to alcohol and drugs. One possible explanation for this could be the diminished ability to cope with the stress inherent in interrogations, associated with substance use disorder and withdrawal (e.g., Gudjonsson et al., 2007). Another important finding was that suspects who showed signs of an ongoing state of intoxication during the interrogations faced more confrontational interrogation techniques by the police compared to suspects who were sober. While these findings are informative and interesting, it is equally important to note that the archival nature of the present study does not allow for any causal inferences regarding the relationship between suspects’ intoxication and substance use disorder, interrogation techniques and confessions.

### Interrogation methods

Regardless of intoxication, our results suggest very few strategic interrogation methods being used by police in this sample with mean domain emphasis scores below 0.5 for *presentation of evidence* and *rapport and relationship building*, and scores below 0.3 for *confrontation/competition* and *emotion provocation.* This might be attributed to the low-stakes nature of the crimes, where an element of routine and “business as usual” is likely. Moreover, the directness of the evidence (e.g., a suspect caught red-handed while driving under the influence) in these cases may make elaborate interrogation tactics superfluous. Other studies that have examined the taxonomy of interrogation methods framework have found a higher degree of use of techniques belonging to the domains, which most likely is due to the fact that these studies have examined high-stakes crimes (e.g., Kelly et al., 2016; Hagsand et al., 2022c). *Presentation of evidence* was the most common domain, which is most likely linked to the aforementioned directness of the evidence. The second most common domain was *rapport and relationship building*, with the most frequently used technique being *ask for open account*, a technique that was added in the main study of the current research. This is good news because letting the suspect share their story uninterrupted improves the gathering of correct information (Leahy-Harland and Bull, 2021).

*Confrontation/competition* was the third most commonly scored domain used by the police. In the context of suspect intoxication, results showed that significantly more confrontational techniques were used with intoxicated than sober suspects. This was surprising as intoxicated suspects were not less cooperative than sober suspects and were not less likely to confess. It is possible that biases or frustration toward intoxicated individuals played a role in interrogators’ use of these techniques, as a recent study on Swedish and Norwegian police found an association between negative emotions among interrogators and the use of confrontational techniques (Magnusson et al., 2021).

For both intoxicated and sober suspects, the techniques *prompt speculation* and *ask the same question repeatedly* were the most frequent. *Prompt speculation* was a new addition to the coding manual in the present study and refers to the interrogator pressuring the suspect into speculating about what might have happened at the crime scene, or what might happen in the future (i.e., what a future blood test will show with respect to intoxication levels). It can, for example, be phrased as *“if you didn’t do it, who do you think did?”* Refusing to answer these questions is often interpreted as a sign of guilt, but if the suspect does answer, that answer can be used against them. In the current sample the prompt speculation tactic almost exclusively concerned the forensic evidence and was phrased as “*what do you think the blood test will show?*” A guilty suspect then has the options of being truthful and confessing to their intoxication/positive test or lying and possibly contradicting the evidence (and thereby having less credibility both regarding subsequent explanations of drug test results and regarding any additional charges). It remains unclear what effect this technique actually has on intoxicated suspects, who might struggle with memory and where inviting speculation could lead to false memories. Previous studies suggest that internalized false confessions can be associated with memory distrust (Gudjonsson et al., 2007). Although little is known about the effects of inviting suspects to speculate on their subsequent statements, past research on the use of speculation and imagination with (vulnerable) *sober witnesses* suggests detrimental effects on the veracity of subsequent statements (e.g., Goff and Roediger, 1998; Schreiber Compo and Parker, 2010). Future research should investigate the effects of this technique in the context of interrogations, including the interrogation of vulnerable suspects under the influence or with substance use disorder.

### Appropriate and inappropriate question types

The frequency of question types compared to strategic interrogation techniques was high in the present sample of interrogations. No significant differences in question types (appropriate versus inappropriate) were found between suspects showing signs of intoxication or not, and there were no differences on this factor between suspects showing signs of substance use disorder and those who were not. This suggests that interrogators did not differentiate between these groups in their choice of questions used. Here too, the high prevalence of appropriate questions could be attributed to the focus on low-stakes crimes where the suspect could be less resistant, thereby avoiding the need for the application of interrogation strategies. That evidence might be more easily accessed in low-stakes crime interrogations related to alcohol and drugs may also be supported by the fact that the *presentation of evidence* domain was the most commonly used domain in the present study.

The results from the present study revealed a high proportion of appropriate question types and a low frequency of inappropriate question types. In contrast to the Kelly and Valencia (2021) study examining United States high-stakes interrogations, the results are striking. In the present study the overall AQD mean scores was 0.75 (Study 1) and 0.85 (Study 2), whereas in Kelly and Valencia’s (2021) study the overall AQD score was 0.24. The difference between high- and low-stakes crimes might explain the variance in AQD score between the studies. It is possible that less resistance from the suspect and less pressure on the interrogator in low-stake crimes may allow for the use of more appropriate question types. However, caution is needed, as it is also possible that the use of written police interrogations in the present study may have contributed to the high proportion of appropriate questions, as it might be easier for the police to summarize many inappropriate questions with just a few (appropriate) open-ended questions when writing down the notes. Other research has found that police investigators sometimes summarize their own questions, just to be able to write down what the interviewee is saying (e.g., Cauchi et al., 2010). With respect to this matter, it is important to note that a Swedish study with juvenile suspects of serious crimes found that most questions asked by the police were inappropriate (Winerdal et al., 2018). Clearly more research is needed on this topic.

### Suspect cooperation and confession rates

Suspects displaying signs of substance use disorder were significantly more cooperative and prone to confess than suspects without indicators of substance use disorder. This might be because alcohol/drug addiction may be risk factors for increased suggestibility and (true/false) confessions (e.g., Pearse et al., 1998; Gudjonsson et al., 2002, 2004). Somewhat surprisingly, suspects who were intoxicated during the interrogation were actually not more prone to cooperate and confess to the crimes compared to sober ones. These findings are similar to the archival United States study of prosecutor case files where alcohol and/or drug intoxicated suspects of serious crimes were just as likely as sober suspects to admit to the crime (Palmer et al., 2013). This might be because acute intoxication does *not always* impact cognitive functions (see Mindthoff et al., 2019, 2021), at least not at low to moderate intoxication levels of alcohol. In the present study, we did not have any information about the actual intoxication levels among the suspects in the interrogations, but future studies could focus on finding ways to sample this information and then analyze it.

### Limitations

Being an archival study with materials provided by the Swedish Police Authority, experimental control over the sample was limited, as is the case in similar archival research (e.g., Kelly et al., 2016). Although specific requests were made when applying for the data, these requests could understandably not always be met. The sample therefore contained a range of different crime types and differed in terms of how many crimes the suspect was interrogated for. However, all suspects were charged with drug- and alcohol-related crimes, therefore fulfilling the main criteria. Due to the lack of control over the received material, a large proportion of the sample had to be excluded based on our excluding criteria (for example repeated interrogations).

It is also important to acknowledge that there are some limitations to coding written documentation from interrogations as some information is lost, such as the eye contact between the police and the suspect which could have provided important additional information about their interaction. In addition, using contemporaneous handwritten or computer-typed notes did not allow us to control for accuracy of these notes, as elaborated on in the introduction and in the method sections. Although electronic recording (audio/video) have been found to be more complete and more accurate compared to notes (Powell et al., 2011) given that investigators can omit some of their own questions to prioritize interviewee information (Cauchi et al., 2010), previous studies have still used contemporaneous notes to study investigative interviews (e.g., Orbach et al., 2012). Future research should try analyzing audio/video recordings of interrogations even if low-stakes interrogations concerning alcohol- and drugs are rarely audio/video recorded (as there currently is not Swedish mandate to do so).

### Implications and future directions

The present set of results suggests that intoxication and/or substance use disorder during the interrogation may render suspects more vulnerable in interrogation settings – a notion that has received little empirical examination thus far. Future research should focus on examining causal relationships, for example in experiments, between suspect intoxication levels and interrogator behavior with an eye toward consequences for true and false confessions and collect data across countries and jurisdictions. It will also be important to examine the effects of intoxication at higher levels, that is, above the legal limit in the context of potential interrogative vulnerability. Examining the impact of breath alcohol level on suspects’ cognition and decision-making is important, in light of recent findings that low to moderate alcohol doses do not affect suggestibility (Mindthoff et al., 2021) or the risk of disclosing transgressions (Mindthoff et al., 2019). Some field studies however suggest that higher intoxication levels may impair cognition among mock suspects (e.g., van Oorsouw et al., 2015).

Although there is a high prevalence of intoxication in various criminal contexts (e.g., Evans et al., 2009; Monds et al., 2021; Hagsand et al., 2022a,b), there is limited research on interrogation procedures with intoxicated suspects and/or suspects suffering from substance use disorder. Previous research has mainly focused on intoxication level at the time of the crime and how this might affect memory of events, mainly in witnesses (e.g., Hagsand et al., 2017; Hildebrand Karlén et al., 2017; Altman et al., 2019; Jores et al., 2019). The coding of intoxication in the current study was based on signs of intoxication and/or substance use disorder *during the interrogation* and not at the actual time of the crime. The suspects’ intoxication status at the time of the crime was not always available in the written interrogations. However, we urge future research to find novel ways to examine how intoxication and substance use status at the *time of the crime* affect subsequent police–suspect interaction and outcomes of the interrogation such as confession or denials, as there is evidence that alcohol/drug status at the time of the crime can affect other legal practitioners such as judges (e.g., Spohn et al., 2014). Also, emerging evidence suggests that intoxicated lab participants may have difficulties in understanding Miranda rights (Mindthoff et al., 2022), with implications for intoxicated suspects’ comprehension of these rights if interrogated in close temporal proximity to the crime.

## Conclusion

To date, interrogators and interviewers must use their own judgment when deciding how to conduct interrogations with intoxicated persons as the research on alcohol/drugs and cognition in the legal context is still limited. Furthermore, many countries lack evidence-based guidelines for interviews with intoxicated and substance using persons (see Evans et al., 2009; Crossland et al., 2018; Monds et al., 2021; Hagsand et al., 2022a,b; Pettersson et al., 2022). As the first novel study on interrogations concerning low-stakes crimes related to alcohol and/or drugs, the present study provides useful information about current Swedish interrogation practices and areas for improvement.

The study concludes that suspects displaying signs of intoxication or substance use disorder may be more vulnerable during police interrogations. Although police mostly asked direct questions instead of using strategic interrogation techniques on suspects, when it came to interrogation techniques, law enforcement used more confrontational techniques in their interactions with intoxicated suspects, compared to sober suspects. Furthermore, suspects displaying signs of substance use disorder were significantly more cooperative and prone to confess than suspects without indicators of substance use disorder. These findings are important but due to the limitations of the present sample, more studies are needed to provide a more detailed understanding of the role of alcohol and drugs in interrogation settings.

## Data availability statement

The datasets presented in this article are not readily available because the lack of ethical permission to share the sensitive and confidential nature of data consisting of police interrogations and the scoring of these. Requests to access the datasets should be directed to AH, angelica.hagsand@psy.gu.se.

## Ethics statement

The studies involving human participants were reviewed and approved by the Swedish National Ethical Review Board (sv. Etikprövningsmyndigheten). Study 1 (Protocol Nos. 2018-158-18 and 2020-00538) and Study 2 (Protocol No. 2020-06960). Written informed consent for participation was not required for this study in accordance with the national legislation and the institutional requirements.

## Author contributions

AH was PI for both Study 1 and 2 and acquired the grant funding, and obtained the ethical permissions and the interrogations from the Swedish Police Authority. AH also had the main responsibility for the manuscript and the progress of the project. HZ and LL archived the raw material digitally, in line with ethical permission due to the sensitive nature of the data and also conducted part of the coding for Study 1 (i.e., the intoxication coding), and all coding for Study 2. After that, they assisted the PI in various tasks of the project (e.g., manuscript, statistical analysis, and editing). CK had the role of expert adviser and contributed with valuable feedback early on in the project concerning conceptual issues, the coding method and the analysis plan, and later on with feedback on the manuscript. NSC was Co-PI and helped to acquire the grant funding, which included feedback on conceptual issues, and later on she also gave valuable feedback on the manuscript. JE contributed early on in the project with feedback on conceptual issues and guidance on which coding framework to use. Later on, JE also contributed with valuable feedback on the manuscript. All authors contributed to the article and approved the submitted version.

## Footnotes

1 Of note, a minority of interrogations in the present study consisted of alcohol- and drug-related crimes in combination with more serious crimes (see Table 1). In Study 1, only one interrogation (2.6%) referred to an alcohol/drug-related crime and aggravated assault/attempted manslaughter. In Study 2, there were nine interrogations (9.3%) that consisted of alcohol/drug related crimes and serious crimes such as aggravated assault/attempted manslaughter, rape/attempted rape, murder/attempted murder. In these instances, it is possible that the interrogations were audio/video recorded and then transcribed by the police. However, it was only explicitly stated in one of these nine interrogations that the interrogation had been recorded. In six of the interrogations, the interrogation had been read out loud and approved, indicating that the written interrogation was based on the interrogator’s notes (in the two remaining interrogations, it was not stated that they were recorded or read out loud).

2
https://osf.io/yqn9v/?view_only=3b7260c8900d437bafa4d4977e58a296

3 For each interrogation, we first obtained the average number of techniques used per domain and block (i.e., 4 domains × 3 blocks = 12): Σ (all techniques)/12 = M_*whole*_. Next, we calculated the average number of within-domain techniques per block: Σ (within-domain techniques)/3 = M_*domain*_. After that, M_*whole*_ and M_*domain*_ were then averaged to create the critical value of M_*combined*_. If the summed within-domain technique scores for a given time block was lower than M_*combined*_, a one (1) was coded as domain emphasis score. If the summed within-domain technique scores for a given time block exceeded M_*combined*_, a two (2) was coded. If no techniques were scored within a domain in a given time block, a zero (0) was coded.

4 According to De Swert (2012), a Krippendorff’s alpha value of α = 0.60 may be acceptable for especially complex codings. Therefore, in this case accounting for the many techniques and question types that were coded, the achieved levels were deemed acceptable.
